# Cytotoxicity and Genotoxicity Assessment of Sandalwood Essential Oil in Human Breast Cell Lines MCF-7 and MCF-10A

**DOI:** 10.1155/2016/3696232

**Published:** 2016-05-11

**Authors:** Carmen Ortiz, Luisa Morales, Miguel Sastre, William E. Haskins, Jaime Matta

**Affiliations:** ^1^Department of Basic Sciences, Divisions of Pharmacology & Toxicology & Cancer Biology, Ponce Health Sciences University School of Medicine, Ponce Research Institute, Ponce, PR 00732, USA; ^2^Public Health Program, Ponce Health Sciences University, Ponce, PR 00732, USA; ^3^Biology Department, University of Puerto Rico at Humacao, Humacao, PR 00792, USA; ^4^Pediatric Biochemistry Laboratory, University of Texas at San Antonio, San Antonio, TX 78249, USA

## Abstract

Sandalwood essential oil (SEO) is extracted from* Santalum* trees. Although *α*-santalol, a main constituent of SEO, has been studied as a chemopreventive agent, the genotoxic activity of the whole oil in human breast cell lines is still unknown. The main objective of this study was to assess the cytotoxic and genotoxic effects of SEO in breast adenocarcinoma (MCF-7) and nontumorigenic breast epithelial (MCF-10A) cells. Proteins associated with SEO genotoxicity were identified using a proteomics approach. Commercially available, high-purity, GC/MS characterized SEO was used to perform the experiments. The main constituents reported in the oil were (Z)-*α*-santalol (25.34%), (Z)-nuciferol (18.34%), (E)-*β*-santalol (10.97%), and (E)-nuciferol (10.46%). Upon exposure to SEO (2–8 *μ*g/mL) for 24 hours, cell proliferation was determined by the MTT assay. Alkaline and neutral comet assays were used to assess genotoxicity. SEO exposure induced single- and double-strand breaks selectively in the DNA of MCF-7 cells. Quantitative LC/MS-based proteomics allowed identification of candidate proteins involved in this response: Ku70 (*p* = 1.37*E* − 2), Ku80 (*p* = 5.8*E* − 3), EPHX1 (*p* = 3.3*E* − 3), and 14-3-3*ζ* (*p* = 4.0*E* − 4). These results provide the first evidence that SEO is genotoxic and capable of inducing DNA single- and double-strand breaks in MCF-7 cells.

## 1. Introduction

Sandalwood essential oil (SEO) is extracted from trees from the* Santalum *genus. Among various species of sandalwood, the most common are the Indian sandalwood (*Santalum album*) and Australian sandalwood (*S. spicatum*) followed by the species found in Hawaii (*S. ellipticum*), New Caledonia (*S. austrocaledonicum*), and French Polynesia (*S. insulare*) [1]. The chemical composition of SEO has been studied in detail. At least 300 chemical constituents have been identified, of which (*Z*)-*α*-santalol and (*Z*)-*β*-santalol are the most abundant [2]. SEO is used in the food industry as a flavoring ingredient and also in the cosmetic and perfume industry [3]. SEO has also been studied as a chemopreventive agent for skin papillomas in mice [4–6] and* in vitro* in human epidermoid carcinoma cells [7]. Matsuo and Mimaki studied the cytotoxicity of various *α*-santalol derivatives and found that some of them present tumor-specific cytotoxicity [8]. This group also found that *α*-santalol induces DNA fragmentation as a result of apoptosis [7]. Even though previous studies have provided valuable data on the pharmacological properties of individual constituents of SEO, there is no information on the activity of whole SEO in human breast cells, or on its DNA-damaging potential. Due to the increased popularity of essential oils (EOs) for massage and aromatherapy, people are frequently exposed to SEO through various routes of administration including the skin. EOs can easily be absorbed through the human skin due to their lipid solubility and extremely low molecular size and the lipophilic nature of the skin itself [9].

DNA damage can be induced by a wide variety of factors such as radiation and chemical substances [10]. While some chemicals can cause DNA damage, mutations are only produced when the DNA repair system of the cell malfunctions or during replication of the damaged DNA [11]. DNA-damaging chemicals are considered genotoxic [12]. Genotoxic compounds can cause mutations in somatic cells that can lead to chromosomal alterations, insertions, deletions, or translocations [13]. Since EOs are present in many household products and are used in folk medicine, an assessment of the potential of these substances to induce damage to DNA is needed. Some groups have begun to evaluate the capacity of EOs to induce DNA damage. For example, Péres and coworkers found that the EO extracted from pariparoba (*Piper gaudichaudianum*), a herb commonly used in Brazilian folk medicine, is cytotoxic and genotoxic against V79 cells [14]. Genotoxicity of pariparoba EO was assessed using the comet and micronucleus assays. This group found that this EO has the capacity to induce DNA damage in a dose-dependent manner. Moreover, they found that lipid peroxidation could be the potential mechanism for its cytotoxic and genotoxic effects [14]. Although the genotoxicity of some EOs has been studied and possible mechanisms have been proposed, there are large gaps in the knowledge of the DNA-damaging potential of EOs in human breast cells. Moreover, there are even fewer studies that elucidate the mechanisms or identify key proteins involved in the induction of DNA damage. Therefore, the objective of this study is to assess the cytotoxic and genotoxic effects of SEO in human breast cell lines MCF-7 and MCF-10A. We also aimed to identify proteins associated with genotoxicity by means of quantitative tandem mass spectrometry-based microwave and magnetic (M^2^) proteomics [15, 16]. This technique allowed us to establish a correlation between relative protein expression and the genotoxic effects of SEO at different exposure times in human breast cancer cells.

## 2. Materials and Methods

### 2.1. Sandalwood Essential Oil

Commercially available 100% pure SEO was acquired from Mountain Rose Herbs Co. (Eugene, OR). Their oils comply with high standards of quality and are certified to be grown using organic components, thus avoiding any interference from pesticides. These oils are certified as organic (through the Oregon Tilth) and are characterized by the company using GC/MS (Table 1).

### 2.2. Liposome Encapsulation for Essential Oil Delivery

EOs, in general, are hydrophobic and biologically unstable as are many other plant products [17]. They have poor solubility in water and are distributed poorly to target sites [18]. Due to these characteristics, we decided to use liposomal encapsulation for delivery to improve the stability and bioavailability of SEO across cell membranes as presented by Shoji and Nakashima (2004) [19]. SEO was encapsulated into liposomes by Ingredient Innovative International (3i) Solutions Company (Wooster, OH). Each liposome is composed of 15% SEO, 78.5% water, 4% enzyme modified lecithin, and 2.5% polysorbate.

### 2.3. Cell Culture

MCF-7 (ATCC® HTB22*™*) and MCF-10A (ATCC® CRL-10317*™*) cells were purchased from American Type Culture Collection (ATCC, MA). MCF-7 cells were maintained in Eagle's Minimum Essential Medium with 10% FBS and 0.01 mg/mL insulin. MCF-10A cells were maintained in Dulbecco's Modified Eagle's Medium with 5% horse serum, 0.5 *µ*g/mL hydrocortisone, 0.01 mg/mL insulin, and 20 ng/mL EGFR. Both cell lines were maintained at 37°C in a humidified incubator containing 5% CO_2_.

### 2.4. MTT Assay

Cell proliferation was measured using the MTT assay as described by Mosmann (1983) [20, 21]. MTT reduction is a measure of mitochondrial activity based on the enzymatic reduction of a tetrazolium salt by the mitochondrial dehydrogenase of viable cells. This provides an estimate of cell viability. MCF-7 and MCF-10A cells were seeded in 96-well plates to a volume of 1,000 cells per well and treated with different concentrations of liposome encapsulated SEO and empty liposomal suspension ranging from 2 to 8 *μ*g/mL for 24 hours. To assess the cytotoxicity of the liposomes, the cells were treated with different volumes of empty liposomes that resembled the amount of liposomes added for each concentration of SEO. None of the volumes of empty liposomes were found to be cytotoxic to the cells (data not shown). After treatment, MTT reagent was dissolved in cell media and added to a final concentration of 1 mg/mL. After four hours, the cells were washed twice with PBS and dissolved in 200 *μ*L of 1x Triton X-100 solution. Absorbance was measured at 570 with a 630 nm background correction using a Synergy HT microplate reader (BioTek, VT). The viability of cells was calculated from the absorbance values. The absorbance of untreated cells was set as 100% viability and the values of treated cells were calculated as percentage of the absorbance of untreated cells.

### 2.5. Alkaline and Neutral Comet Assay

**Figure 1:**
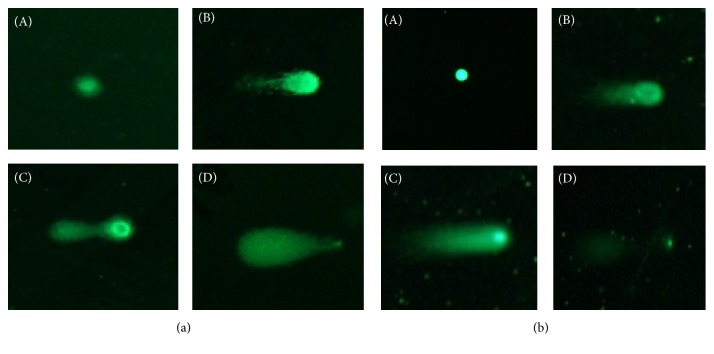
Representation of the different comet classes in the alkaline (a) and neutral (b) comet assay. Cells were visually scored according to tail length into four classes: (A) class 1: undamaged, with no tail, (B) class 2: with tail shorter than the diameter of the head (nucleus), (C) class 3: with tail as long as 1-2x the diameter of the head, and (D) class 4: with tail longer than 2x the diameter of the head.

Using the information obtained in the cytotoxicity studies and the IC_50_ values for each cell line, we selected the concentrations of 2, 4, 6, and 8 *µ*g/mL of SEO for performing the alkaline and neutral comet assays. Comet assay experiments were performed using the CometAssay kit (Trevigen, MD). After treatment with 2, 4, 6, and 8 *μ*g/mL of SEO for 24 hours, MCF-7 and MCF-10A cells were trypsinized (0.05%) and centrifuged at 200 ×g for 10 min. An aliquot of the cell suspension was mixed with 500 *μ*L of Comet LMAgarose and spread on CometSlides. Slides were placed at 4°C for 30 minutes and then immersed in lysis solution at 4°C overnight. For the alkaline comet assay, slides were incubated for 20 min in alkaline unwinding solution (200 mM NaOH and 1 mM EDTA) at room temperature. Electrophoresis was performed in the CometAssay ES II unit (Trevigen, MD) at 21 V for for 30 min. Slides were washed twice with water and incubated in 70% ethanol for 5 min. For the neutral comet assay, after lysis, the slides were incubated for 30 min in neutral electrophoresis buffer (100 mM Tris Base and 300 mM sodium acetate). Electrophoresis was performed at 21 V for 45 min. Slides were then immersed in DNA precipitation solution (1 M ammonium acetate) for 30 min and finally in 70% ethanol for 30 min. After drying, the slides were stained with Yoyo-1 (Life Technologies, CA) and analyzed using an Olympus BX-60 fluorescence microscope equipped with an excitation filter (BP 510 nm). Ethyl methanesulfonate (EMS) (Sigma, MO) was used as a positive control at a concentration of 12 mM for 4 hrs. Fifty cells per slide were analyzed for DNA migration. Cells were visually scored according to tail length into four classes: (1) class 1, undamaged, with no tail; (2) class 2, with tail shorter than the diameter of the head (nucleus); (3) class 3, with tail as long as 1-2x the diameter of the head; and (4) class 4, with tail longer than 2x the diameter of the head (Figure 1). The DNA damage index (DI) was calculated as described by Sastre et al. (2005), using a modified weighted average formula:

(1) $$\begin{array}{c} DI=\frac{\sum RN}{\sum N}, \end{array}$$

where *R* refers to the damage category (from 1 to 4) and *N* is the number of cells belonging to each damage category [22]. Kobayashi et al. (1995) showed that the manual microscopic analysis was less time-consuming and had equal or better sensitivity than the computerized image analysis [23].

### 2.6. Microwave and Magnetic (M^2^) Sample Preparation

Sample preparation for isobaric labeling was performed as described by Raphael et al. (2014) [24]. C8 magnetic beads (BcMg, Bioclone Inc., CA) were suspended in 1 mL of 50% methanol. Around 100 *µ*L of the beads was washed with equilibration buffer (200 mM NaCl and 0.1% trifluoroacetic acid (TFA)). Lysate from breast cancer cell lines untreated (1_126_) and treated with 6 *µ*g/mL SEO for 5 minutes (1_127_), 1 hour (1_128_), 5 hours (1_129_), and 24 hours (1_130_); and a pooled reference of all the samples (1_131_) (25–100 *µ*g at 1 *µ*g/*µ*L) was mixed with preequilibrated beads and 1/3rd sample binding buffer (800 mM NaCl and 0.4% TFA) by volume. The beads were washed twice with 40 mM triethylammonium bicarbonate (TEAB), and 10 mM dithiothreitol (DTT) was added followed by microwave heating for 10 s. After removing the DTT solution, 50 mM iodoacetamide (IAA) was added followed by microwave heating for 10 s. Beads were washed with 40 mM TEAB and resuspended in 150 *μ*L of 40 mM TEAB.* In vitro* proteolysis was performed with 4 *μ*L of trypsin in a 1 : 25 trypsin-to-protein ratio (stock = 1 *μ*g/*μ*L in 50 mM acetic acid) and microwave-heated for 20 s in triplicate. The supernatant was transferred to a new tube for immediate use or stored at −80°C. Released tryptic peptides from digested lysates, including the reference material described above, were modified at the N-terminus and at lysine residues with the tandem mass tagging (TMT)-6plex isobaric labeling reagents (Thermo Scientific, CA). Each sample was encoded with one of the TMT-126-130 reagents, while reference material was encoded with the TMT-131 reagent. Then, 41 *μ*L of anhydrous acetonitrile was added to 0.8 mg of TMT labeling reagent and 25 *µ*g of lysate was added and microwave-heated for 10 s. To quench the reaction, 8 *μ*L of 5% hydroxylamine was added to the sample at room temperature. To normalize across all SEO-treated samples, TMT-encoded lysates from individual samples, labeled with the TMT-126-130 reagents, respectively, were mixed with the reference material encoded with the TMT-131 reagent in a 1_126_ : 1_127_ : 1_128_ : 1_129_ : 1_130_ : 1_131_ ratio in single sample mixture that was stored at −80°C until further use.

### 2.7. Capillary Liquid Chromatography-Fourier Transform-Tandem Mass Spectrometry (LC-FT-MS/MS) with Protein Database Searching

Capillary LC-FT-MS/MS was performed as described by Raphael et al. (2014) [24] using a splitless nanoLC-2D pump (Eksigent, CA), a 50 *µ*m-i.d. column packed with 7 cm of 3 *µ*m-o.d. C18 particles, and a hybrid linear ion trap-Fourier-transform tandem mass spectrometer (LTQ-ELITE; Thermo Fisher, CA). The reverse-phase gradient was 2 to 62% of 0.1% formic acid (FA) in acetonitrile over 60 min at 350 nL/min. The top six most abundant eluting ions were fragmented by data-dependent high-energy collision-induced dissociation (HCD). Probability-based and error-tolerant protein database searching of MS/MS spectra against the Trembl protein database (release of 2013) were performed with a 10-node Mascot cluster (version 2.3.02, Matrix Science, London, UK). Search criteria included peak picking with Mascot Distiller, 10 ppm precursor ion mass tolerance, 0.8 Da product ion mass tolerance, 3 missed cleavages, trypsin, carbamidomethyl cysteines and oxidized methionines as variable modifications, an ion score threshold of 20, and TMT-6-plex for quantification.

### 2.8. Pathway Analysis

Ingenuity Pathways Analysis software (IPA, Ingenuity® Systems) was used to perform the biochemical pathway analysis of the samples under study, according to the suggestions of the manufacturer. Mascot results were imported to the software and a core analysis was performed for each data file. Proteins with differential expression related to genes in the IPA knowledge base were mapped onto the canonical signaling pathways. The resulting histogram provided by IPA presented the percentage of proteins that were quantified in each canonical signaling pathway. Each pathway was inspected for signal pathway enrichment, where *p* values were assigned by IPA.

### 2.9. Western Blotting

Expression of candidate proteins involved in DNA double-strand breaks (DSBs), cell cycle regulation upon DNA damage, and cellular metabolism were validated by Western blotting. 1.00 × 10^6^ human breast cancer cells were seeded in 60 mm tissue culture plates and treated with 6 *µ*g/mL of SEO for 24 hours. Cells were harvested at different exposure times (5 min, 1 hr, 5 hrs, and 24 hrs) and lysed with RIPA buffer supplemented with PhosSTOP phosphatase inhibitor cocktail (Roche Applied Science, IN). Upon centrifugation, total cellular proteins were collected for quantification using the Quick Start Bradford Protein Assay (Bio-Rad, CA). 15 *µ*g of the total cellular proteins from each sample was treated with *β*ME (5% by volume) prior to boiling for 5 minutes and separating proteins on 10% SDS-PAGE gels. Separated proteins were transferred to a 0.45 *µ*m nitrocellulose membrane (Bio-Rad, CA) followed by blocking with 5% milk in TTBS for 2 hours. The membrane was washed with TTBS buffer for 5 min in triplicate and probed with primary rabbit or mouse antibodies: [(1 : 1000) anti-Ku70 (D35), (1 : 1000) anti-14-3-3*ζ*/*δ* (D7H5) (Cell Signaling, MA)], [(1 : 500) anti-Ku80 (3D8) monoclonal antibody (EpiGentek, NY)], or [(1 : 2000) anti-epoxide hydrolase (ab96774) antibody (Abcam, MA)] in TTBS overnight. After 90 min of incubation with (1 : 2500) anti-rabbit or anti-mouse IgG HRP-conjugated secondary antibody (Jackson ImmunoResearch Laboratories, PA), immunoreactive protein bands were detected using the VisiGlo Prime HRP Chemiluminescent Substrate Kit (AMRESCO, OH). Images were captured using the ChemiDoc*™* XRS+ System (Bio-Rad, CA) with Quantity One® imaging software (Bio-Rad, CA).

### 2.10. Statistical Analysis

Results are reported as the mean ± SEM of three independent experiments. The significance of differences was estimated using Student's paired *t*-test. The difference was considered statistically significant when the *p* value was less than 0.05. Asterisks denote statistical significance: (*∗*) *p* < 0.05 and (*∗∗*) *p* < 0.01. As for the results obtained through M^2^ proteomics, candidate proteins were selected from the IPA software analysis based on their *p* values and their biological relevance. Fold change values were analyzed using One-Sample *t*-test to determine statistical significance. All statistical analyses were performed using the GraphPad Prism version 6.0 (GraphPad Software, CA).

## 3. Results

### 3.1. Cytotoxicity Assay

**Figure 2:**
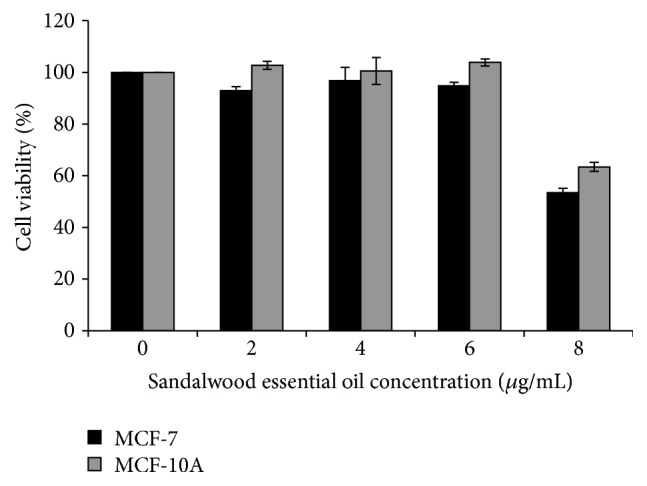
Sandalwood essential oil reduces cell viability in MCF-7 and MCF-10A cells with increasing concentration. Dose-response curve shows sandalwood essential oil cytotoxicity to MCF-7 and MCF-10A cells within the concentration interval of 2–8 *µ*g/mL. Values presented are the mean of three independent experiments in triplicate (mean ± SEM).

After treatment of the MCF-7 and MCF-10A cell lines with eight concentrations of SEO, there was a decrease in cell viability to less than 20% in both cell lines (Figure 2). From these data, we calculated IC_50_, which is the concentration of SEO required to reduce cell viability by 50%. For the MCF-7 cell line, IC_50_ was 8.03 *µ*g/mL, while for the MCF-10A cell line it was 12.3 *µ*g/mL.

### 3.2. Genotoxicity Assessment

#### 3.2.1. DNA Single-Strand Breaks Caused by Sandalwood Essential Oil

**Figure 3:**
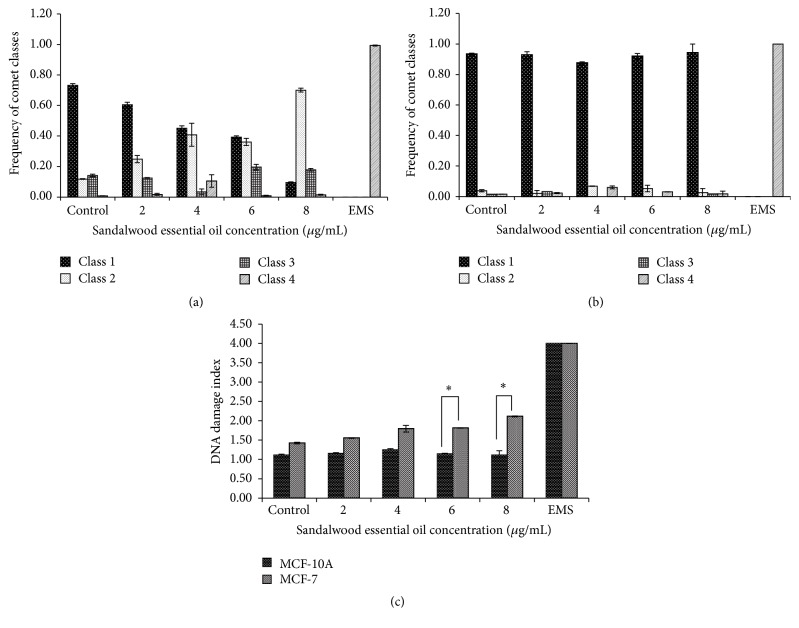
Distribution of comet classes (DNA damage) in (a) MCF-7 and (b) MCF-10A cells. (c) DNA damage was calculated as DNA damage index. Increasing concentration of sandalwood essential oil induces single-strand breaks in MCF-7 cells. However, the same effect is not seen in MCF-10A cells. Every bar represents the mean ± SEM of three independent experiments. Asterisk (*∗*) denotes statistical significance *p* < 0.05 between the two cell lines upon treatment (Student's *t*-test).

**Figure 4:**
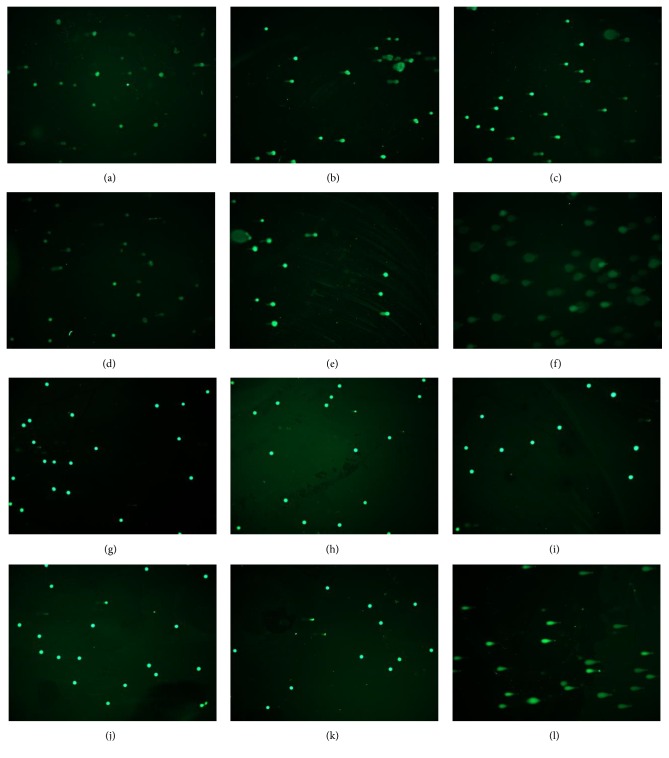
Alkaline comet assay performed in MCF-7 ((a)–(f)) and MCF-10A ((g)–(l)) cells after exposure to sandalwood essential oil for 24 hours at concentrations of ((b), (h)) 2 *µ*g/mL, ((c), (i)) 4 *µ*g/mL, ((d), (j)) 6 *µ*g/mL, and ((e), (k)) 8 *µ*g/mL. EMS (12 mM) was used as a positive control ((f), (l)). Panels (a) and (g) show untreated cells.

The alkaline version of the comet assay allowed us to determine the capacity of SEO of inducing DNA single-strand breaks (SSBs) in both cell lines. Our results show that increasing concentration of SEO reduces the incidence of class 1 comets to almost 10% and increases the frequency of class 2 comets in MCF-7 cells (Figures 3(a) and 4(a)–4(e)). In Figure 3(c), it can be seen that the DI increases in a dose-dependent manner. The highest DIs were observed at 6 and 8 *µ*g/mL with values of 1.81 and 2.11, respectively. This effect was statistically significant for both concentrations (*p* = 0.039 and 0.030, resp.) (Figure 3(c)). For the human breast nontumorigenic cell line MCF-10A, this effect was not observed. Even with increasing concentration of SEO, the incidence of class 1 comets remained consistent and the appearance of class 2, class 3, or class 4 comets was rare (Figures 3(b) and 4(g)–4(k)). In Figure 3(c), the DI remains almost unchanged even at the highest concentration of SEO. When comparing the DI in the two cell lines at the same concentrations, the increase in DNA damage is more evident at 6 and 8 *µ*g/mL of SEO. At 6 *µ*g/mL, the DI for MCF-10A cells was 1.14 whereas for MCF-7 cells it was 1.81 (*p* = 0.01). At 8 *µ*g/mL, the DI for MCF-10A cells was 1.11, while for MCF-7 cells it was 2.11 (*p* = 0.05).

#### 3.2.2. DNA Double-Strand Breaks Caused by Sandalwood Essential Oil

**Figure 5:**
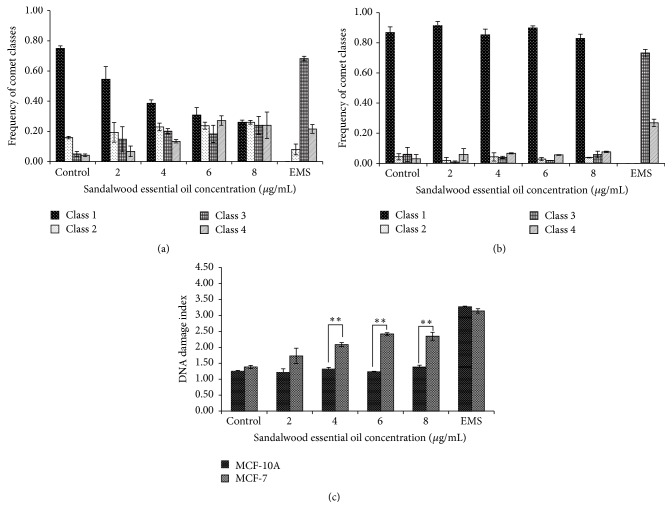
Distribution of comet classes (DNA damage) in (a) MCF-7 and (b) MCF-10A cells. (c) DNA damage was calculated as DNA damage index. Sandalwood essential oil induces double-stranded breaks in the DNA of MCF-7 cells. However, the same effect is not seen in MCF-10A cells. Every bar represents the mean ± SEM of three independent experiments. Asterisks (*∗∗*) denote statistical significance *p* < 0.01 between the two cell lines upon treatment (Student's *t*-test).

**Figure 6:**
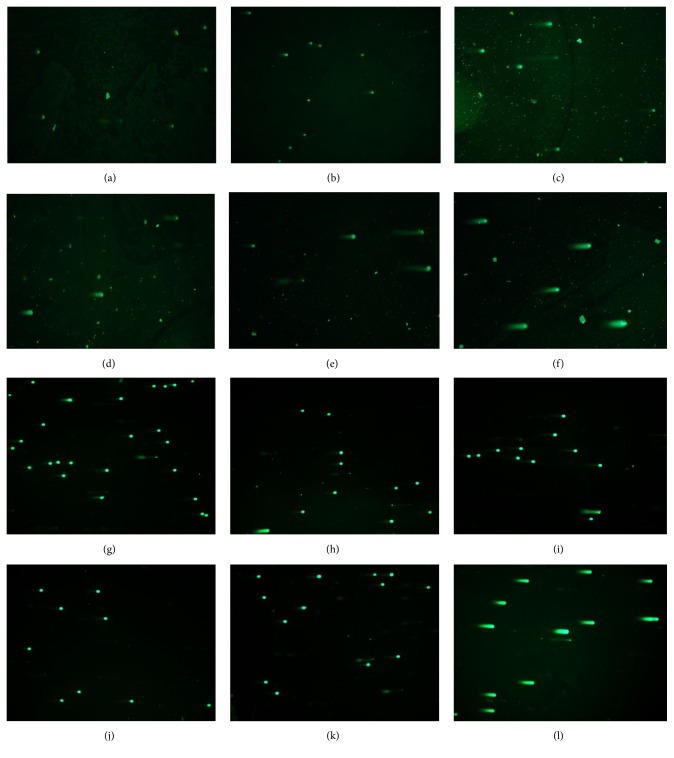
Neutral comet assay performed in MCF-7 ((a)–(f)) and MCF-10A ((g)–(l)) cells after exposure to sandalwood essential oil for 24 hours at concentrations of ((b), (h)) 2 *µ*g/mL, ((c), (i)) 4 *µ*g/mL, ((d), (j)) 6 *µ*g/mL, and ((e), (k)) 8 *µ*g/mL. EMS (12 mM) was used as a positive control ((f), (l)). Panels (a) and (g) show untreated cells.

The neutral comet assay allows for determination of DNA double-strand breaks (DSBs). In these experiments, a similar trend was observed as in the alkaline comet assay (Figures 3 and 4). As presented in Figures 5(a) and 6(a)–6(e), SEO decreased the appearance of class 1 comets to almost 30% with increasing concentration in MCF-7 cells, therefore, increasing the appearance of higher class comets 3 and 4 (Figure 5(a)). At the highest concentration studied (8 *µ*g/mL SEO), the frequency of comets was almost the same for all four classes, therefore, yielding a higher DI (Figure 5(a)). While control (untreated) MCF-7 cells had a DI of 1.38, as the concentration of SEO increased the DI also increased. Similar to the results of the alkaline comet assay, at 6 and 8 *µ*g/mL, the DIs were 2.42 and 2.35, respectively (*p* = 0.02, *p* = 0.05) (Figure 5(c)). For MCF-10A cells, the results were similar to the ones obtained through the alkaline comet assay. The frequency of class 1 comets remained almost unchanged with increasing concentration of SEO, while the appearance of higher class comets was rare (Figure 5(b)). As a result of this, the DI remained similar independent of the concentration of SEO used (Figure 5(c)). When comparing the two cells lines, the greatest difference in DI was at the concentrations of 4, 6, and 8 *µ*g/mL. At 4 *µ*g/mL, the DI for MCF-10A cells was 1.32, while for MCF-7 cells it was 2.09 (*p* = 0.008). At 6 *µ*g/mL, the DI for MCF-10A cells was 1.23, while for MCF-7 cells it was 2.42 (*p* = 0.03). At the highest concentration studied (8 *µ*g/mL SEO), the DI for MCF-10A cells was 1.38, while for MCF-7 cells it was 2.35 (*p* = 0.04) (Figure 5(c)).

### 3.3. Proteomics Analysis of Proteins Associated with Sandalwood Essential Oil Genotoxicity

Quantitative tandem mass spectrometry-based proteomics at multiple time points incorporating rapid M^2^ sample preparation allowed us to establish a correlation between relative protein expression and the genotoxic effects of SEO in MCF-7 cells. Of the pool of 130 proteins that were differentially expressed in MCF-7 cells upon exposure to SEO, we selected proteins involved in DNA repair pathways, cell cycle regulation, and cellular metabolism including Ku70 (*p* = 1.37*E* − 2), Ku80 (*p* = 5.8*E* − 3), EPHX1 (*p* = 3.3*E* − 3), and 14-3-3*ζ* (*p* = 4.0*E* − 4) (Table 2).

**Figure 7:**
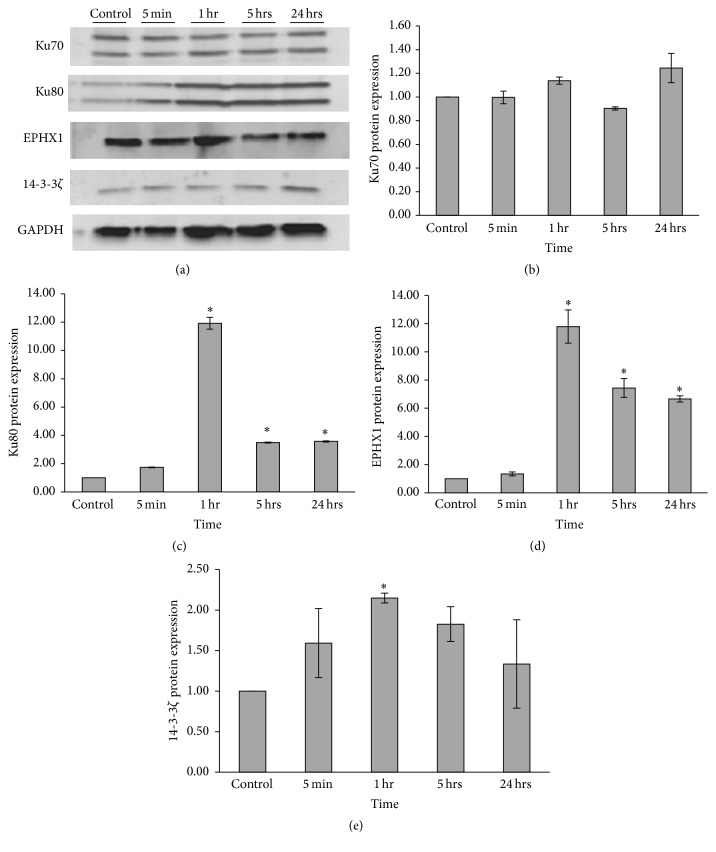
Sandalwood essential oil induces protein expression of Ku70, Ku80, EPHX1, and 14-3-3*ζ* in MCF-7 cells. (a) Western blot analysis from 25 *µ*g of protein extracted from MCF-7 cells treated with SEO for 5 min, 1 hr, 5 hrs, and 24 hrs. GAPDH was used as loading control. Densitometric quantification of (b) Ku70 shows the highest induction of the protein at 24 hours, while (c) Ku80, (d) EPHX1, and (e) 14-3-3*ζ* achieve their highest induction at 1 hr of exposure. Asterisk (*∗*) denotes statistical significance *p* ≤ 0.05 when compared with the control (Student's *t*-test). Each bar represents the mean of three independent experiments (mean ± SEM).

The expression of selected candidate proteins was confirmed with Western blotting.  Figure 7 shows the results obtained for this validation. Almost all of the proteins studied were induced after 1 hour of exposure to SEO. However, their expression profile varies depending on the exposure time. For example, Ku70 shows a slight increase in expression at 1 hour of exposure but reaches its peak at 24 hours (Figure 7(b)). However, this increase was not statistically significant (*p* = 0.290). In contrast, Figure 7(c) shows that Ku80 has its greater expression after 1 hour of exposure to SEO (*p* = 0.024). After this time point, the expression of this protein becomes reduced but it is still greater than that in the untreated sample. EPHX1 has an expression profile similar to the one of Ku80 with the highest expression after 1 hour of exposure to SEO (*p* = 0.050) and decreases in expression at 5 and 24 hours (*p* = 0.050 and 0.025, resp.) (Figure 7(d)). 14-3-3*ζ* also becomes induced at 1 hour of exposure to the oil (*p* = 0.030) (Figure 7(e)).

## 4. Discussion

Our results suggest that SEO has selective genotoxic effects in MCF-7 cells when compared with noncancerous MCF-10A cells at noncytotoxic concentrations. Our findings provide evidence that SEO is capable of inducing single- and double-strand DNA breaks in the human breast cancer cell line MCF-7. This study provides, to our knowledge, the first evidence that SEO has dose-dependent cytotoxic and genotoxic effects in the human breast adenocarcinoma cell line (MCF-7), whereas it was cytotoxic but not genotoxic to the MCF-10A cell line. Only a few studies have used the MCF-10A cell line as a model to study genotoxicity in nontumorigenic breast epithelial cells. A study by Stankevicins and coworkers studied the genotoxic effect of low dose radiation in this cell line using three doses of X-ray radiation including 12 and 48 mGy/28 kV and 5 Gy/30 kV. After radiation exposure, the cells were allowed to recover for 4 and 24 hours and the DNA damage was measured using the comet assay. This group found that although irradiation increased the amount of DNA lesions initially in MCF-10A cells, at 24 hours, the cells recovered their DNA integrity as was observed in the reduced levels of DNA damage measured with the comet assay [25]. This finding is also consistent with a study by Francisco and coworkers, in which they studied the induction and processing of DNA damage in breast cancer cells and the nontumorigenic cell line MCF-10A, upon exposure to radiotherapy-relevant *γ*-radiation doses. They assessed DNA damage using the comet assay to measure single- and double-strand breaks. Their results show that breast cancer cells (MCF-7) have a tendency to accumulate more DNA lesions than MCF-10A after *γ*-radiation exposure [26]. These studies provide evidence on the decreased susceptibility of MCF-10A cells to DNA damage. These findings, previously reported in the literature, can partially explain the selective genotoxicity of SEO.

Our study also examines the question of potential genotoxic effects caused by the whole SEO rather than on specific chemical components such as *α*-santalol. When studying EOs, some biological effects are attributed to their main constituents; however, the possible synergy of all of the chemical components in the mixture working together must also be evaluated. In our study, we have shown that SEO induces DNA damage in the form of single- and double-strand breaks at nontoxic concentrations in MCF-7 cells. Since we assessed the genotoxic capacity of SEO through the comet assay, we decided to use quantitative LC/MS-based proteomics at multiple time points, incorporating rapid M^2^ sample preparation, to identify specific proteins related to DNA double-strand breaks (DSBs), cell cycle control and regulation upon DNA damage, and cellular metabolism of genotoxic compounds.

In the proteomics data analysis,* Ku70* and* Ku80* were found to be differentially expressed upon SEO exposure. These proteins are involved in the process of repairing DNA DSBs; therefore, their differential expression correlates with the results of the alkaline and neutral comet assay. Upon induction of DNA DSBs, the cell can activate two types of repair: homologous recombination or nonhomologous end joining (NHEJ). NHEJ allows the ligation of two DNA ends without requiring sequence homology [27]. One of the key components of this DNA repair pathway is the Ku protein which is a heterodimeric complex composed of Ku70 (70 kDa) and Ku80 (80 kDa) subunits. This complex binds selectively to double-stranded DNA ends in a sequence independent manner. Ku70 and Ku80 initiate the repair process of DNA DSBs by activation of the DNA-dependent protein kinase after binding to the DNA DSBs [28]. Gu et al. (1997) showed that cells deficient in Ku70 expression have increased radiosensitivity and defects in DNA end-binding activity [29]. Moreover, Ku80 null mice have shown an increase in chromosomal aberrations and malignant transformations [30]. Upregulation of Ku70 occurs upon exposure to ionizing radiation via p53/ATM-dependent mechanism [31]. Various studies have suggested that the Ku complex recognizes DSBs and serves as an alignment factor that promotes end joining [32–35]. The first step for DSB repair is the recognition of the damage by sensor proteins like the ATP-dependent helicase II (Ku70). This enzyme is found in increased levels 30 minutes after DSB induction [36]. Both of these proteins were found to be induced in samples treated with SEO, after 1 hour of exposure.

Our results also show a differential expression of* EPHX1*, or the human microsomal epoxide hydrolase (mEH). This protein is one of the many biotransformation enzymes which functions in the detoxification of chemical epoxide intermediates produced during phase I oxidation reactions [37]. mEH actively metabolizes potentially carcinogenic or genotoxic epoxides, such as those derived from the oxidation of polyaromatic hydrocarbons [38]. Epoxides are highly reactive compounds with an electrophilic functional group. This electrophilic group allows the epoxide to react with electron-rich moieties in the DNA and produce DNA adducts or DNA strand breaks [39]. Styrene, for example, can be activated to a genotoxic intermediate in the human body. The genotoxic intermediate, an epoxide, becomes inactivated by the mEH [40]. Several alkene epoxides have genotoxic effects causing DNA damage when evaluated using the comet assay [41]. It is possible that some components of SEO might be causing the production of epoxides that also induce DNA damage. Upon a genotoxic insult, the cell needs to stop its replication to allow the DNA repair enzymes to identify and repair the damage. The differential expression of* 14-3-3ζ* suggests that DNA repair is occurring after exposure to SEO. 14-3-3 proteins regulate cell division and play an important role in stopping cell cycle progression after the DNA damage checkpoints are activated [42]. Dirksen et al. (2006) studied protein expression in 14 human lymphoblast cell lines after induction of DSBs using bleomycin. 14-3-3*ζ*, a protein involved in cell cycle regulation, was found among the proteins that were expressed in the samples [36]. Upon induction of DNA damage, 14-3-3*ζ* binds to Cdc25 and removes it from the nucleus, halting the cell cycle [43]. Stopping cell cycle progression is crucial to prevent replication of damaged DNA and to activate the machinery needed for DNA repair.

## 5. Conclusion

The capacity of SEO to induce single- and double-strand breaks in human breast adenocarcinoma cells was confirmed by the alkaline and neutral comet assays. Although this assay does not provide information on specific DNA repair pathways involved in this process, the use of proteomics allowed us to define more precisely the possible DNA repair pathways and proteins that are being induced upon the DNA damage caused by SEO on breast cell lines.

Here, we present a possible mechanistic explanation for the genotoxic response of MCF-7 cells to SEO found in our experiments. Ku70/80 induction provides evidence that the DSB repair system becomes activated. However, the cell is not able to effectively repair the DSBs, possibly due to the amount of damage induced by SEO. We have also found evidence that some of the DSBs could be caused due to epoxide formation due to the induction of EPHX1. The cell cycle is halted for DSB repair due to the activity of the 14-3-3 family, mostly because of 14-3-3*ζ* activity. We provide evidence that, upon the genotoxic insult of SEO exposure, the cell is capable of activating several pathways to activate DNA repair. However, in the case of MCF-7 cells, this activation was not sufficient to mitigate the effects of SEO since although the proteins were induced, DSBs were still present as was revealed by the comet assay. Future studies will focus on the assessment of other genotoxicity endpoints such as chromosomal aberrations upon SEO exposure in MCF-7 cells using the micronucleus assay and investigating the status of these proteins in nontumorigenic breast epithelial cells to which SEO did not cause single- or double-strand breaks. In conclusion, our findings on the genotoxic potential of SEO in breast cancer cells could lead to potential discoveries of molecules with specific anticancer activity that have a selective genotoxic effect to breast cancer cells. This project could be the first step in the process of finding alternative therapies with less toxicity to noncancerous cells.

## Figures and Tables

**Table 1:** Chemical composition of *Santalum austrocaledonicum* essential oil analyzed by GC/MS.

Compound	Percent (%)
1-Furfurylpyrrole	0.11
7-Epi-sesquithujene	0.08
*α*-Santalene	0.67
Trans-*α*-bergamotene	0.11
Epi-*β*-santalene	0.43
*β*-Acoradiene	0.13
*γ*-Curcumene	0.27
*α*-Curcumene	0.28
Helifolen-12-al	0.07
*β*-Bisabolene	0.21
*β*-Curcumene	0.54
*β*-Sesquiphellandrene	0.14
8,14-Cedranoxide	0.09
(E)-Nerolidol	0.53
Dendrolasin	0.38
Sesquiterpenoids	2.66
*β*-Bisabolol	1.60
(Z)-*α*-Santalol	25.34
*α*-Santalal	0.95
(Z)-Trans-*α*-Bergamotol	4.35
8-Cedren-13-ol	1.83
(Z)-Epi-*β*-Santalol	4.07
(Z,Z)-Farnesol	1.56
(E)-*β*-Santalol	10.97
(Z)-Nuciferol	18.34
(E)-*α*-Santalol	0.48
(Z)-*α*-Cis-Bergamotol	0.76
(E)-Nuciferol	10.46
(Z)-Lanceol	7.34
(Z)-*α*-Trans-bergamotol acetate	1.33
(E)-*α*-Trans-bergamotol acetate	0.59
(E)-Lanceol acetate	0.83
(E)-*β*-Santalol acetate	0.15
1,3-Hydroxy-bisabola-2,10-diene	0.57

Total	98.22

**Table 2:** Fold changes in protein expression obtained from the Ingenuity Pathway Analysis software for Ku70, Ku80, EPHX1, and 14-3-3*ζ* in MCF-7 cells upon treatment with sandalwood essential oil at four exposure times compared to the control.

Protein	Exposure time to sandalwood essential oil					*p* value
	Control	5 min	1 hr	5 hrs	24 hrs	
Ku70	0.916	1.429	2.07	2.432	2.120	1.37*E* − 02
Ku80	1.767	4.048	4.147	5.848	5.124	5.80*E* − 03
EPXH1	2.040	4.31	4.323	5.663	4.828	3.30*E* − 03
14-3-3*ζ*	0.599	2.631	2.408	2.170	2.626	4.00*E* − 04

## References

[1] Harbaugh D. T., Baldwin B. G. (2007). Phylogeny and biogeography of the sandalwoods (*Santalum*, Santalaceae): repeated dispersals throughout the Pacific. *American Journal of Botany*.

[2] Hasegawa T., Izumi H., Tajima Y., Yamada H. (2012). Structure-odor relationships of *α*-santalol derivatives with modified side chains. *Molecules*.

[3] Burdock G. A., Carabin I. G. (2008). Safety assessment of sandalwood oil (*Santalum album* L.). *Food and Chemical Toxicology*.

[4] Dwivedi C., Abu-Ghazaleh A. (1997). Chemopreventive effects of sandalwood oil on skin papillomas in mice. *European Journal of Cancer Prevention*.

[5] Dwivedi C., Guan X., Harmsen W. L. (2003). Chemopreventive effects of *α*-santalol on skin tumor development in CD-1 and SENCAR mice. *Cancer Epidemiology Biomarkers and Prevention*.

[6] Dwivedi C., Zhang Y. (1999). Sandalwood oil prevents skin tumour development in CD1 mice. *European Journal of Cancer Prevention*.

[7] Kaur M., Agarwal C., Singh R. P., Guan X., Dwivedi C., Agarwal R. (2005). Skin cancer chemopreventive agent, *α*-santalol, induces apoptotic death of human epidermoid carcinoma A431 cells via caspase activation together with dissipation of mitochondrial membrane potential and cytochrome c release. *Carcinogenesis*.

[8] Matsuo Y., Mimaki Y. (2012). *α*-Santalol derivatives from Santalum album and their cytotoxic activities. *Phytochemistry*.

[9] Keville K., Green M. (1995). The sense of smell. *Aromatherapy: A Complete Guide to the Healing Art*.

[10] Clancy S. (2008). DNA damage & repair: mechanisms for maintaining DNA integrity. *Nature Education*.

[11] Brown T. A. (2002). Mutation, repair and recombination. *Genomes*.

[12] FDA (2008). *S2(R1) Genotoxicity Testing and Data Interpretation for Pharmaceuticals Intended for Human Use*.

[13] Preston R. J., Hoffman G. R. (2013). Genetic toxicology. *Casarett & Doull's Toxicology: The Basic Science of Poisons*.

[14] Péres V. F., Moura D. J., Sperotto A. R. M. (2009). Chemical composition and cytotoxic, mutagenic and genotoxic activities of the essential oil from *Pipergaudichaudianum* Kunth leaves. *Food and Chemical Toxicology*.

[15] Raphael I., Mahesula S., Kalsaria K. (2012). Microwave and magnetic (M^2^) proteomics of the experimental autoimmune encephalomyelitis animal model of multiple sclerosis. *Electrophoresis*.

[16] Mahesula S., Raphael I., Raghunathan R. (2012). Immunoenrichment microwave and magnetic proteomics for quantifying CD47 in the experimental autoimmune encephalomyelitis model of multiple sclerosis. *Electrophoresis*.

[17] Liolios C. C., Gortzi O., Lalas S., Tsaknis J., Chinou I. (2009). Liposomal incorporation of carvacrol and thymol isolated from the essential oil of *Origanum dictamnus* L. and in vitro antimicrobial activity. *Food Chemistry*.

[18] Ortan A., Campeanu G., Dinu-Pirvu C., Popescu L. (2009). Studies concerning the entrapment of *Anethum graveolens* essential oil in liposomes. *Romanian Biotechnological Letters*.

[19] Shoji Y., Nakashima H. (2004). Nutraceutics and delivery systems. *Journal of Drug Targeting*.

[20] Mosmann T. (1983). Rapid colorimetric assay for cellular growth and survival: application to proliferation and cytotoxicity assays. *Journal of Immunological Methods*.

[21] Denizot F., Lang R. (1986). Rapid colorimetric assay for cell growth and survival. Modifications to the tetrazolium dye procedure giving improved sensitivity and reliability. *Journal of Immunological Methods*.

[22] Sastre M. P., Collado E., Collazo G. (2005). DNA damage in the Caribbean mussel Brachiodontes exustus: a comet assay evaluation. *Bulletin of Marine Science*.

[23] Kobayashi H., Sugiyama C., Morikawa Y., Hayashi M., Sofuni T. (1995). A comparison between manual microscopic analysis and computerized image analysis in the single cell gel electrophoresis assay. *MMS Communications*.

[24] Raphael I., Mahesula S., Purkar A. (2014). Microwave & magnetic (M^2^) proteomics reveals CNS-specific protein expression waves that precede clinical symptoms of experimental autoimmune encephalomyelitis. *Scientific Reports*.

[25] Stankevicins L., Almeida da Silva A. P., Ventura dos Passos F. (2013). MiR-34a is up-regulated in response to low dose, low energy X-ray induced DNA damage in breast cells. *Radiation Oncology*.

[26] Francisco D. C., Peddi P., Hair J. M. (2008). Induction and processing of complex DNA damage in human breast cancer cells MCF-7 and nonmalignant MCF-10A cells. *Free Radical Biology and Medicine*.

[27] Jeggo P. A. (1998). DNA breakage and repair. *Advances in Genetics*.

[28] Morio T., Kim H. (2008). Ku, Artemis, and ataxia-telangiectasia-mutated: signalling networks in DNA damage. *International Journal of Biochemistry and Cell Biology*.

[29] Gu Y., Jin S., Gao Y., Weaver D. T., Alt F. W. (1997). Ku70-deficient embryonic stem cells have increased ionizing radiosensitivity, defective DNA end-binding activity, and inability to support V(D)J recombination. *Proceedings of the National Academy of Sciences of the United States of America*.

[30] Difilippantonio M. J., Zhu J., Chen H. T. (2000). DNA repair protein Ku80 suppresses chromosomal aberrations and malignant transformation. *Nature*.

[31] Brown K. D., Lataxes T. A., Shangary S. (2000). Ionizing radiation exposure results in up-regulation of Ku70 via a p53/ataxia-telangiectasia-mutated protein-dependent mechanism. *The Journal of Biological Chemistry*.

[32] Cary R. B., Peterson S. R., Wang J., Bear D. G., Bradbury E. M., Chen D. J. (1997). DNA looping by Ku and the DNA-dependent protein kinase. *Proceedings of the National Academy of Sciences of the United States of America*.

[33] Feldmann E., Schmiemann V., Goedecke W., Reichenberger S., Pfeiffer P. (2000). DNA double-strand break repair in cell-free extracts from Ku80-deficient cells: implications for Ku serving as an alignment factor in non-homologous DNA end joining. *Nucleic Acids Research*.

[34] Nick McElhinny S. A., Snowden C. M., McCarville J., Ramsden D. A. (2000). Ku recruits the XRCC4-ligase IV complex to DNA ends. *Molecular and Cellular Biology*.

[35] Ramsden D. A., Geliert M. (1998). Ku protein stimulates DNA end joining by mammalian DNA ligases: a direct role for Ku in repair of DNA double-strand breaks. *The EMBO Journal*.

[36] Dirksen E. H. C., Cloos J., Braakhuis B. J. M., Brakenhoff R. H., Heck A. J. R., Slijper M. (2006). Human lymphoblastoid proteome analysis reveals a role for the inhibitor of acetyltransferases complex in DNA double-strand break response. *Cancer Research*.

[37] Fretland A. J., Omiecinski C. J. (2000). Epoxide hydrolases: biochemistry and molecular biology. *Chemico-Biological Interactions*.

[38] Hosagrahara V. P., Rettie A. E., Hassett C., Omiecinski C. J. (2004). Functional analysis of human microsomal epoxide hydrolase genetic variants. *Chemico-Biological Interactions*.

[39] Oesch F., Hengstler J. G., Arand M. (2004). Detoxication strategy of epoxide hydrolase-the basis for a novel threshold for definable genotoxic carcinogens. *Nonlinearity in Biology, Toxicology, Medicine*.

[40] Oesch F. (1973). Mammalian epoxide hydrases: inducible enzymes catalysing the inactivation of carcinogenic and cytotoxic metabolites derived from aromatic and olefinic compounds. *Xenobiotica*.

[41] Fabiani R., Rosignoli P., De Bartolomeo A., Fuccelli R., Morozzi G. (2012). Genotoxicity of alkene epoxides in human peripheral blood mononuclear cells and HL60 leukaemia cells evaluated with the comet assay. *Mutation Research—Genetic Toxicology and Environmental Mutagenesis*.

[42] Meek S. E. M., Lane W. S., Piwnica-Worms H. (2004). Comprehensive proteomic analysis of interphase and mitotic 14-3-3-binding proteins. *The Journal of Biological Chemistry*.

[43] Qi W., Martinez J. D. (2003). Reduction of 14-3-3 proteins correlates with increased sensitivity to killing of human lung cancer cells by ionizing radiation. *Radiation Research*.

